# Generation of Insulin-Producing Cells from Canine Adipose Tissue-Derived Mesenchymal Stem Cells

**DOI:** 10.1155/2020/8841865

**Published:** 2020-10-18

**Authors:** Takahiro Teshima, Keiji Okamoto, Kazuho Dairaku, Tomokazu Nagashima, Masaki Michishita, Ryohei Suzuki, Hirotaka Matsumoto, Hidekazu Koyama

**Affiliations:** ^1^Laboratory of Veterinary Internal Medicine, Department of Veterinary Clinical Medicine, School of Veterinary Medicine, Faculty of Veterinary Science, Nippon Veterinary and Life Science University, 1-7-1 Kyonan-cho, Musashino, Tokyo 180-8602, Japan; ^2^Research Center for Animal Life Science, Nippon Veterinary and Life Science University, 1-7-1 Kyonan-cho, Musashino, Tokyo 180-8602, Japan; ^3^Laboratory of Veterinary Pathology, Department of Veterinary Pathobiology, School of Veterinary Medicine, Faculty of Veterinary Science, Nippon Veterinary and Life Science University, 1-7-1 Kyonan-cho, Musashino, Tokyo 180-8602, Japan

## Abstract

The potential of mesenchymal stem cells (MSCs) to differentiate into nonmesodermal cells such as pancreatic beta cells has been reported. New cell-based therapy using MSCs for diabetes mellitus is anticipated as an alternative treatment option to insulin injection or islet transplantation in both human and veterinary medicine. Several protocols were reported for differentiation of MSCs into insulin-producing cells (IPCs), but no studies have reported IPCs generated from canine MSCs. The purpose of this study was to generate IPCs from canine adipose tissue-derived MSCs (AT-MSCs) *in vitro* and to investigate the effects of IPC transplantation on diabetic mice *in vivo*. Culturing AT-MSCs with the differentiation protocol under a two-dimensional culture system did not produce IPCs. However, spheroid-like small clusters consisting of canine AT-MSCs and human recombinant peptide *μ*-pieces developed under a three-dimensional (3D) culture system were successfully differentiated into IPCs. The generated IPCs under 3D culture condition were stained with dithizone and anti-insulin antibody. Canine IPCs also showed gene expression typical for pancreatic beta cells and increased insulin secretion in response to glucose stimulation. The blood glucose levels in streptozotocin-induced diabetic mice were decreased after injection with the supernatant of canine IPCs, but the hyperglycemic states of diabetic mice were not improved after transplanting IPCs subcutaneously or intramesenterically. The histological examination showed that the transplanted small clusters of IPCs were successfully engrafted to the mice and included cells positive for insulin by immunofluorescence. Several factors, such as the transplanted cell number, the origin of AT-MSCs, and the differentiation protocol, were considered potential reasons for the inability to improve the hyperglycemic state after IPC transplantation. These findings suggest that canine AT-MSCs can be differentiated into IPCs under a 3D culture system and IPC transplantation may be a new treatment option for dogs with diabetes mellitus.

## 1. Introduction

Diabetes mellitus (DM) is a global concern not only in human medicine but also in veterinary medicine. DM is a major endocrine disease in dogs, and dogs with DM show similarities to patients with type 1 DM, which is classified as insulin-dependent diabetes. Owners of dogs with DM need to inject dogs with insulin every day. In humans, islet transplantation is one of the therapeutic options for type 1 DM [1, 2]. However, islet transplantation has several limitations for widespread clinical use even in human medicine [3, 4] and is unfeasible in veterinary medicine. The field of regenerative medicine has progressed, and many researchers are aiming to establish new cell-based therapies for various diseases [5–7]. Transplantation of insulin-producing cells (IPCs) differentiated from stem cells is anticipated as an alternative to insulin injection or islet transplantation as a treatment option for DM patients. Several studies have reported the successful differentiation of pluripotent stem cells such as induced pluripotent stem cells (iPS) and embryonic stem cells (ES) into IPCs [8–11]. Some groups have attempted to differentiate multipotent stem cells such as mesenchymal stem cells (MSCs) into IPCs, and some results for potential cell-based therapies for DM have been reported [12–14]. Adipose tissue-derived MSCs (AT-MSCs) are an ideal source for IPC transplantation for veterinary medicine, because adipose tissue is abundant and can be obtained with minimally invasive procedures. The aim of this study was to perform differentiation of canine AT-MSCs into IPCs and investigate the effects of IPC transplantation on diabetic mice.

## 2. Materials and Methods

This research is composed of two studies. Study 1 performed the generation of IPCs from canine AT-MSCs *in vitro*. Study 2 conducted a functional evaluation of the canine IPCs on streptozotocin-induced diabetic mice. All experimental protocols involving the use of dogs and mice were approved by the Bioethics Committee at Nippon Veterinary and Life Science University.

### 2.1. Isolation and Expansion of AT-MSCs

Adipose tissue was aseptically collected from falciform ligament fat of three anaesthetized dogs (males; mean age, 1.5 years; mean body weight, 10.4 kg). The tissue was washed extensively in phosphate buffer solution (PBS), minced, and digested with collagenase type I (Sigma-Aldrich) at 37°C for 45 min with intermittent shaking. After washing with PBS and centrifuging, the pellets containing the stromal vascular fraction were resuspended, filtered through a 100 *μ*m nylon mesh, and incubated overnight in Dulbecco's modified Eagle's medium (DMEM) supplemented with 10% fetal bovine serum (FBS; Nichirei Bioscience) and a 1% antibiotic-antimycotic solution (Thermo Fisher Scientific) in a humidified atmosphere with 5% CO_2_ at 37°C. Unattached cells were removed by changing the medium, and the attached cells were washed twice with PBS. Thereafter, the medium was replaced every 3-4 days. At 80-90% confluence, the cells were detached with trypsin-EDTA solution (Sigma-Aldrich) and passaged repeatedly.

### 2.2. Phenotype Analysis of Canine AT-MSCs

Passage 2 AT-MSCs were analyzed by flow cytometry [15, 16]. Cells (2 × 10^5^ cells) were placed in fluorescence-activated cell sorting (FACS) tubes (BD Biosciences), washed with FACS buffer (PBS containing 2% FBS), and then incubated with the following fluorescein- (FITC-) or phycoerythrin- (PE-) conjugated antibodies: anti-CD14-FITC (BD Pharmingen), anti-CD29-PE (BioLegend), anti-CD34-PE (R&D Systems), anti-CD44-PE (BioLegend), anti-CD45-FITC (eBioscience), and anti-CD90-PE (eBioscience) or their respective isotype controls. The cells were washed twice with FACS buffer and resuspended in 500 *μ*l of FACS buffer. Fluorescence was evaluated by flow cytometry in a FACSCalibur instrument (BD Biosciences). Data were analyzed using WinMDI 2.9 analysis software.

### 2.3. Differentiation of Canine AT-MSCs into IPCs

Differentiation was performed according to a previously reported protocol [17], with modifications. Canine AT-MSCs at passage 3 were seeded in conventional 6-well attachment plates (1.5 × 10^6^ cells/well) or 96-well ultralow attachment plates (Thermo Fisher Scientific) with or without *μ*-pieces (cellnest *μ*-piece, Fujifilm) (1.0 × 10^5^ cells/well with or without *μ*-pieces of 0.05 mg/well) as 3D culture. AT-MSCs were cultured using step 1 (from day 0 to day 2) medium containing FBS-free DMEM with high glucose (25 mM) and 0.5 mM *β*-mercaptoethanol (Sigma-Aldrich). At day 3, small clusters that formed in the 96-well ultralow attachment plates were transferred into 6-well ultralow attachment plates (Corning). Next, cells were cultured in step 2 (from day 3 to day 10) medium containing FBS-free DMEM with high glucose (25 mM), 1% glutamine supplement (GlutaMAX, Thermo Fisher Scientific), 1% nonessential amino acid (FUJIFILM Wako), 20 ng/ml of recombinant human basic fibroblast growth factor (FUJIFILM Wako), 20 ng/ml of recombinant human epidermal growth factor (FUJIFILM Wako), 1% N2 supplement (Thermo Fisher Scientific), 1% B27 supplement (Thermo Fisher Scientific), and 10 nM exendin-4 (Sigma-Aldrich). Finally, cells were cultured in step 3 (from day 11 to day 21) medium containing FBS-free DMEM with high glucose (25 mM), 1% N2 supplement, 1% B27 supplement, 10 nM exendin-4, 10 mM nicotinamide (FUJIFILM Wako), 10 ng/ml betacellulin (Sigma-Aldrich), 50 ng/ml recombinant human activin-A (FUJIFILM Wako), and 50 ng/ml recombinant human hepatocyte growth factor (FUJIFILM Wako).

### 2.4. Dithizone (DTZ) Staining

DTZ (Sigma-Aldrich) staining was performed according to a previously reported protocol [17, 18]. DTZ stock solution was prepared with 50 mg of DTZ in 5 ml of dimethyl sulfoxide and stored at -20°C. For staining, 10 *μ*l of the stock solution was added to 1 ml of serum-free DMEM and filtered through a 0.2 *μ*m nylon filter; this solution was used as the DTZ working solution. Cells at the end of the differentiation protocol were incubated in the DTZ working solution at 37°C for 30 min. Cells were examined with a stereomicroscope after the plates were rinsed three times with PBS.

### 2.5. Quantitative RT-PCR

Total RNA from differentiated cells was extracted using a TRIzol Plus RNA Purification Kit according to the manufacturer's instructions (Thermo Fisher Scientific). cDNA was synthesized from 1 *μ*g of total RNA using random primers and the GoScript Reverse Transcriptase System (Promega), according to the manufacturer's instructions. Real-time RT-PCR analyses were performed using the SYBR Green Real-Time PCR Master Mix (Promega) to determine the mRNA levels of pancreatic duodenal homeobox (Pdx-1), neurogenin 3 (Ngn3), and glucose transporter 2 (GLUT2). Glyceraldehyde 3-phosphate dehydrogenase (GAPDH) mRNA level was used for normalization. The primer sequences were as follows: Pdx-1—forward 5′-CCTACGTTGCAGAGCCAGAA-3′ and reverse 5′-GTGCCTCTCGGTCAAGTTCA-3′; Ngn3—forward 5′-GAGCGCAATCGAATGCACAA-3′ and reverse 5′-TAGAGGCTGTGGTCCGCTAT-3′; GLUT2—forward 5′-CCCACAACCCCATGCCTAAT-3′ and reverse 5′-ATCCCCTAGCCATCCACCAA-3′; and GAPDH—forward 5′-GATGGGCGTGAACCATGAG-3′ and reverse 5′-TCATGAGGCCCTCCACGAT-3′. Amplification conditions were 95°C for 2 min, followed by 40 cycles of 95°C for 15 sec and 60°C for 60 sec. After 40 cycles, a dissociation curve was generated to verify the specificity of each primer. All reactions were performed in duplicate. Expression levels of target genes were normalized to the level of GAPDH and quantified by the *ΔΔ*Ct method.

### 2.6. Glucose-Stimulated Insulin Secretion Assay

To determine the *in vitro* ability of the differentiated AT-MSCs, the insulin secretory response to glucose was measured using a previously reported method [12, 17, 19], with modifications. The differentiated cells (1.5 × 10^6^ AT-MSCs) were preincubated for 1 h in glucose-free Krebs-Ringer bicarbonate (KRB). The cells were then incubated with KRB containing 5.5 mM or 25 mM of glucose for 2 h. The KRB media were collected and frozen at -80°C until evaluation. The insulin level was measured using a canine insulin ELISA kit (FUJIFILM Wako Shibayagi).

### 2.7. Immunofluorescence

The small clusters including *μ*-pieces formed from the 3D culture system (study 1) and transplanted IPCs (study 2) were fixed in 4% paraformaldehyde, dehydrated, and embedded in paraffin; samples were cut into 4 *μ*m sections. The sections were dewaxed in xylene, hydrated, and blocked with a commercial blocking reagent (UltraCruz Blocking Reagent, Santa Cruz) for 1 h in a humidified chamber at room temperature. Then, sections were incubated with a monoclonal insulin antibody conjugated with Alexa Flour 488 (1 : 100 dilution; 2D11-H5, Santa Cruz) overnight at 4°C in a humidified chamber. After washing with PBS containing 0.05% of Tween 20, sections were covered with mounting medium with DAPI (VECTASHIELD, H-1200, Vector Laboratories). Immunofluorescence images were captured using a multipurpose microscope (BZ-X700, Keyence).

### 2.8. STZ-Induced Diabetic Mice

Twenty-five male C57BL/6JJcl mice (B6, 5-6 weeks) and fifteen male immunodeficient mice (Crlj:SHO-*Prkdc^scid^Hr^hr^*; SHO, 5-6 weeks) were used in study 2. The mice were housed in a temperature-controlled and light-controlled room (12 h light/dark cycles) and allowed free access to water and standard laboratory food.

To induce a diabetic state in the mice, 200 mg/kg of streptozotocin (STZ, Sigma-Aldrich) was administrated intraperitoneally as a single injection. The diabetic states were confirmed after two consecutive measurements of blood glucose level over 350 mg/dl or one measurement over 400 mg/dl with a glucometer.

### 2.9. Effect of Insulin Secreted from Canine IPC on STZ-Induced Diabetic B6 Mice

Twenty-one B6 mice confirmed with the diabetic status were divided into four treatment groups: group I: 200 *μ*l of PBS (*n* = 5), group II: 200 *μ*l of FBS-free DMEM (*n* = 5), group III: 200 *μ*l of the supernatant of undifferentiated AT-MSC culture medium (*n* = 5), and group IV: 200 *μ*l of the supernatant of canine IPC culture medium (containing 1 ng of canine insulin as measured by ELISA) (*n* = 6). All mice were kept in fasting condition (with free access to water) for 12 h after intraperitoneal injection with the appropriate solution, and blood glucose levels were measured at 0, 3, 6, 9, and 12 h after injection.

The blood glucose levels of group IV mice were reevaluated as the same time points with alternative injection with 200 *μ*l of FBS-free DMEM intraperitoneally after 2 days from administration of the supernatant of IPC culture medium.

### 2.10. IPC Transplantation to STZ-Induced Diabetic SHO Mice

Twelve mice confirmed with diabetic status were divided into four groups (*n* = 3 mice/group): group I (control): intraperitoneally injected with PBS, group II: intraperitoneally injected with 1.5 × 10^6^ undifferentiated AT-MSCs, group III: transplanted with small clusters of IPCs into the inguinal subcutaneous region, and group IV: transplanted with small clusters of IPCs into the intramesentery. In groups III and IV, mice were transplanted with small clusters of IPC formed from 3D culture with *μ*-pieces (30 wells of 96-well ultralow attachment plates) under anesthesia with a mixture of medetomidine (0.75 mg/kg), midazolam (4 mg/kg), and butorphanol (5 mg/kg). Nonfasting blood glucose levels were measured at days 3, 7, 10, and 15 after injection or transplantation. Transplanted mice were sacrificed at day 15 after blood collection for measurement of canine insulin concentrations, and the transplanted IPC clusters were harvested for histological analysis.

### 2.11. Statistical Analysis

All data are presented as the mean ± standard deviation. Differences between two groups were compared with the Welch *t*-test. Differences among multiple groups were assessed by one-way or two-way analysis of variance, and differences were compared using the Tukey-Kramer post hoc test. *P* < 0.05 was considered statistically significant. Statistical analyses were performed using Excel 2019 with add-in software Statcel 3.

## 3. Results

### 3.1. Characterization of AT-MSCs

AT-MSCs from three beagles were successfully cultured and expanded as described in Materials and Methods. The majority of the cells expressed the mesenchymal stem cell markers CD29 (97.58 ± 1.14%), CD44 (99.54 ± 0.25%), and CD90 (96.23 ± 0.97%), and very few expressed CD14 (1.38 ± 0.08%), CD34 (0.68 ± 0.06%), or CD45 (1.13 ± 0.08%).

### 3.2. Generation of IPCs from Canine AT-MSCs *In Vitro* (Study 1)

#### 3.2.1. DTZ Staining

**Figure 1:**
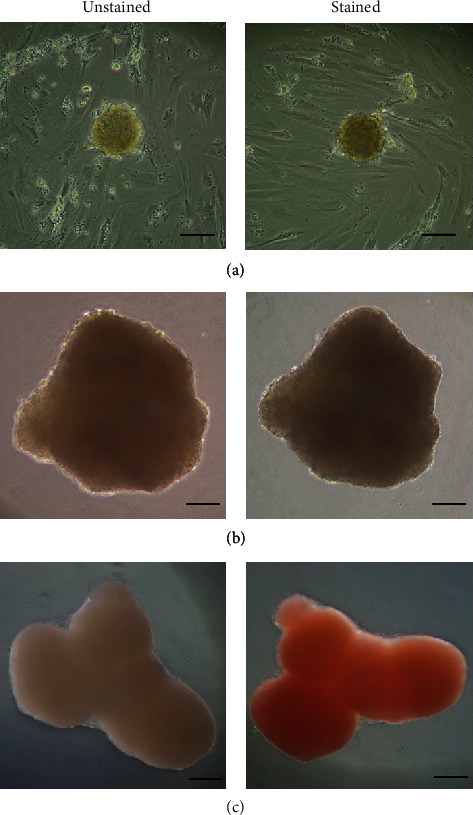
Dithizone staining of canine adipose tissue-derived mesenchymal stem cells at the end of *in vitro* differentiation. (a) Cells cultured in the differentiated protocol using the two-dimensional culture system using an attachment plate. (b) Undifferentiated adipose tissue-derived mesenchymal stem cells using the three-dimensional culture system with *μ*-pieces. (c) Differentiated adipose tissue-derived mesenchymal stem cells using the three-dimensional culture system with *μ*-pieces. Bar = 200 *μ*m.

Differentiated AT-MSCs under conventional culture conditions (cultured in attachment dish) were not stained with DTZ, while differentiated AT-MSCs cultured under 3D conditions with or without *μ*-pieces were stained with DTZ (Figure 1).

#### 3.2.2. Gene Expression

**Figure 2:**
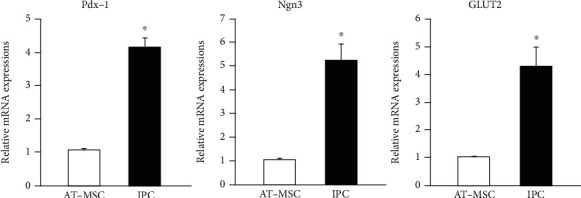
Expression of endocrine pancreatic marker genes in undifferentiated and differentiated adipose tissue-derived mesenchymal stem cells. Pdx-1, Ngn3, and GLUT2 mRNA expressions in insulin-producing cells (IPC) generated under the three-dimensional culture system with *μ*-pieces were 4.1, 5.2, and 4.3 times higher, respectively, than those in undifferentiated adipose tissue-derived mesenchymal stem cells (AT-MSCs). Data are expressed as the mean ± standard deviation. ^∗^*P* < 0.05 vs. AT-MSC.

To clarify the characteristic features of the differentiated AT-MSCs cultured under 3D condition with *μ*-pieces, the gene expression of endocrine pancreatic markers was assessed using quantitative RT-PCR. The expressions of Pdx-1, Ngn3, and GLUT2 mRNAs were significantly higher in differentiated AT-MSCs under 3D condition with *μ*-pieces compared with levels in undifferentiated AT-MSCs (*P* < 0.05) (Figure 2).

#### 3.2.3. Glucose-Stimulated Insulin Secretion

**Figure 3:**
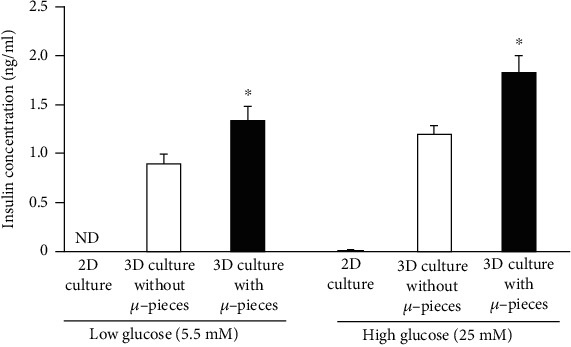
*In vitro* insulin release in response to glucose stimulation. The insulin secretion ability under glucose stimulation was higher in the IPCs generated by the 3-dimensional (3D) culture system with *μ*-pieces than in cells cultured without *μ*-pieces. Data are expressed as the mean ± standard deviation. ^∗^*P* < 0.05 vs. 3D culture without *μ*-pieces. ND: not detected.

We next evaluated the insulin secretion from canine IPC in response to glucose (Figure 3). Low levels of insulin were detected in the supernatant of differentiated AT-MSCs cultured under 2D condition when stimulated with high glucose. Otherwise, the differentiated AT-MSCs under 3D condition with or without *μ*-pieces secreted insulin when stimulated with low glucose (with *μ*-pieces, 1.18 ± 0.10 ng/ml; without *μ*-pieces, 0.89 ± 0.10 ng/ml). The differentiated AT-MSCs under 3D condition with *μ*-pieces secreted significantly higher insulin levels compared with cells cultured without *μ*-pieces (*P* < 0.05). Differentiated AT-MSCs cultured under 3D condition with and without *μ*-pieces secreted higher amounts of insulin in response to high glucose concentrations compared with low glucose (with *μ*-pieces, 1.88 ± 0.17 ng/ml; without *μ*-pieces, 1.24 ± 0.14 ng/ml).

#### 3.2.4. Immunofluorescence Staining

**Figure 4:**
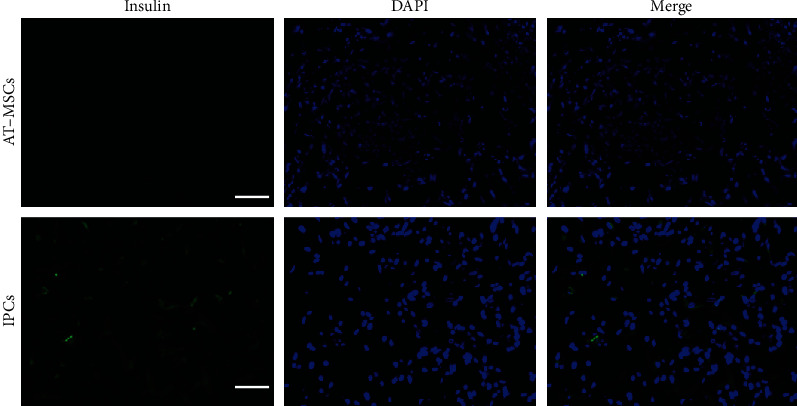
Immunofluorescence of canine adipose-derived mesenchymal stem cells (AT-MSCs) following *in vitro* differentiation into insulin-producing cells (IPCs). Cells were stained for insulin (green) with counterstaining for DAPI (blue). Bar = 50 *μ*m.

Immunofluorescence analysis was performed to investigate the expression of insulin in AT-MSCs. Differentiated AT-MSCs under 3D condition showed positive cytoplasmic staining for insulin, while undifferentiated AT-MSCs were negative for insulin (Figure 4).

### 3.3. Functional Assessment of the IPCs from Canine AT-MSCs *In Vivo* (Study 2)

#### 3.3.1. Effect of Insulin Secreted from Canine IPC on STZ-Induced Diabetic B6 Mice

**Figure 5:**
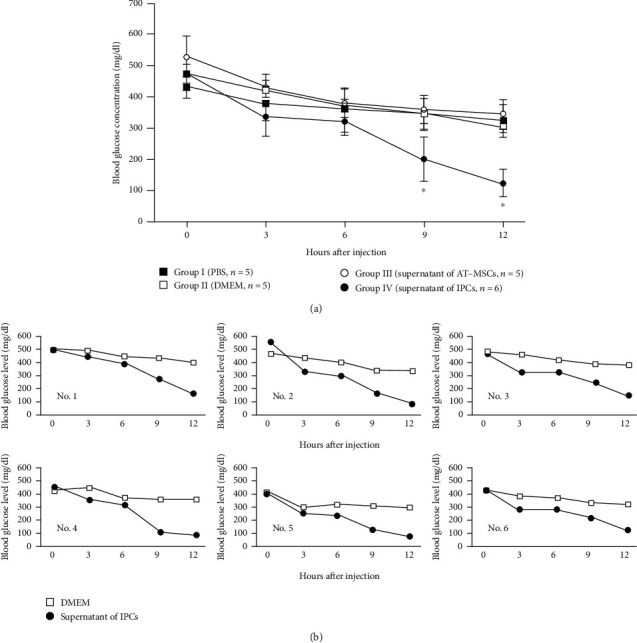
Blood glucose levels in diabetic mice after injection with various treatments. (a) Comparison among groups. The blood glucose levels at 9 and 12 h after injection in group IV (supernatant of IPCs) were significantly lower compared with those in groups I (PBS), II (DMEM), and III (supernatant of AT-MSCs). Data are expressed as the mean ± standard deviation. ^∗^*P* < 0.05 vs. groups I, II, and III. (b) Comparison of the blood glucose levels in the same mice after injection with the supernatant of IPCs and DMEM. The blood glucose levels were lower after injection with the supernatant of IPCs in all six mice.

STZ-induced diabetic mice were kept in fasting condition after intraperitoneal injection with each solution. The blood glucose levels of groups I (PBS), II (DMEM), and III (supernatant of ADSC culture medium) gradually decreased after 12 h observation (group I, 433 mg/dl to 322 mg/dl; group II, 473 mg/dl to 303 mg/dl; and group III, 527 mg/dl to 343 mg/dl). In contrast, the blood glucose levels of group IV (supernatant of IPC culture medium) at 0 h were 472 mg/dl, but after administration of the supernatant of IPCs, the levels significantly decreased compared with those of groups I, II, and III; levels at 3, 6, 9, and 12 h were 336, 322, 200, and 123 mg/dl, respectively (*P* < 0.05) (Figure 5(a)).

Group IV mice were injected with 200 *μ*l of FBS-free DMEM, and blood glucose levels were observed during 12 h after 2-day administration of the supernatant of IPCs. The blood glucose concentrations of all mice were not remarkably decreased after injection with DMEM (Figure 5(b)).

#### 3.3.2. IPC Transplantation to STZ-Induced Diabetic SHO Mice

**Figure 6:**
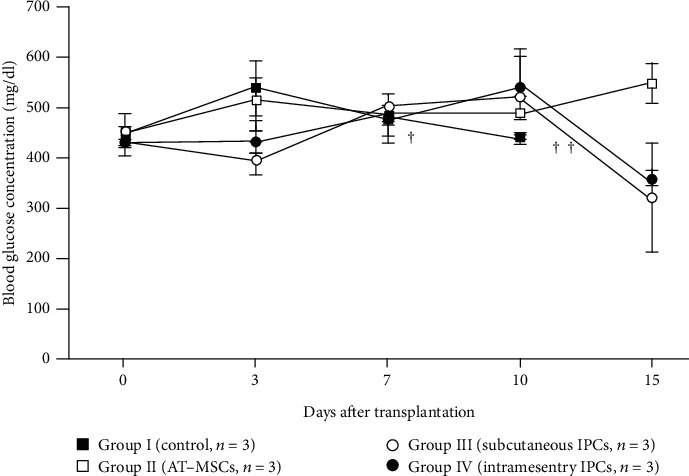
Nonfasting blood glucose levels after transplantation. The blood glucose levels at day 15 were decreased in groups III (subcutaneous IPC transplantation) and IV (intramesentery IPC transplantation) compared with group II (AT-MSC transplantation), but the hyperglycemic state was not improved. †: death in group IV.

Blood glucose concentrations were monitored during 15 days after IPC subcutaneous or intramesenteric transplantation. All mice of groups II (undifferentiated AT-MSC transplantation), III (subcutaneous transplantation), and IV (intramesenteric transplantation) were alive over the 15 days in the observation period, but all three mice in group I (control) died at day 7 (*n* = 1) and day 13 (*n* = 2). The blood glucose level of the mouse in group III was decreased to 171 mg/dl at day 15; however, both group III and IV mice had a continued hyperglycemic state after IPC transplantation (mean blood glucose levels at day 15 were 546 mg/dl, 320 mg/dl, and 359 mg/dl in groups II, III, and IV, respectively) (Figure 6). Serum canine insulin concentrations in all groups at day 15 were not detected by a canine insulin ELISA.

#### 3.3.3. Histopathology of the Transplanted IPCs

**Figure 7:**
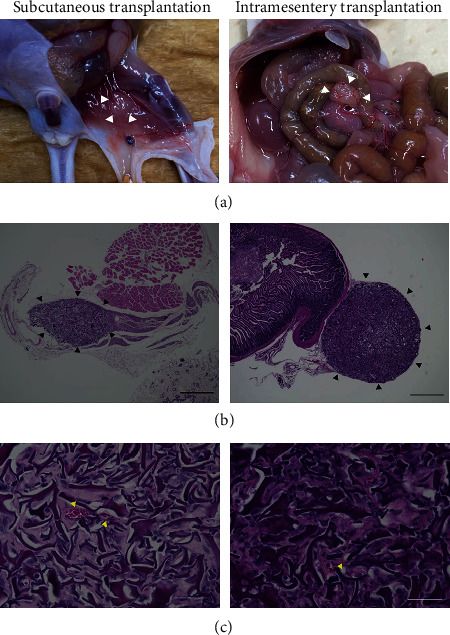
Transplanted small clusters of IPCs at subcutaneous and intramesentery sites. (a) Images at autopsy. The microvessels ran through the surface of both subcutaneous and intramesentery transplanted small clusters of IPCs (white arrowheads). IPCs engrafted in the transplanted sites in the mice. (b) Hematoxylin-eosin (HE) staining in the transplanted small clusters of IPCs (black arrowheads). Bar = 500 *μ*m. (c) Necrosis in the central location of small clusters of IPCs was not observed, but angiogenesis (yellow arrowheads) was confirmed in HE staining. Bar = 50 *μ*m.

**Figure 8:**
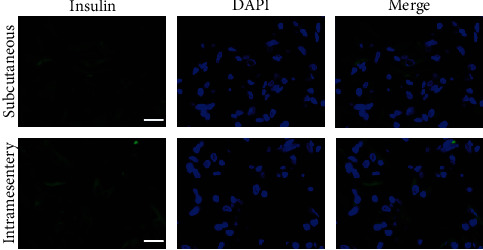
Immunofluorescence of transplanted small clusters of IPCs in diabetic mice. Small clusters of IPCs showed positive insulin staining (green) in both subcutaneous transplantation and intramesentery transplantation. Bar = 50 *μ*m.

We next examined the transplanted IPCs at subcutaneous and intramesentery sites on day 15 posttransplantation. Blood vessels were present at the surface of IPC clusters, and IPC clusters seemed to be successfully engrafted at transplanted sites. Light microscopic imaging showed vascularization of inner IPC clusters, and central necrosis of IPCs was not observed (Figure 7). Both subcutaneous and intramesenteric transplanted IPC clusters showed positive insulin staining by immunofluorescence (Figure 8).

## 4. Discussion

Adipose tissue is an abundant source of MSCs, and AT-MSCs can differentiate toward adipogenic, chondrogenic, and osteogenic cell lineages. Recent studies showed that AT-MSCs have the potential to differentiate into nonmesodermal cells such as neural cells, hepatocytes, and pancreatic beta cells [20–24]. Therefore, new strategies for the treatment of DM using AT-MSCs that differentiated into IPCs have been actively researched [25, 26]. In the veterinary field, canine DM is a common endocrine disease, and currently, the only available treatment option for canine DM is insulin injection throughout life.

To establish a new treatment option for canine DM as an alternative to daily insulin injection, we performed differentiation of canine AT-MSCs into IPCs *in vitro* (study 1) and examined the function of the generated IPCs from canine AT-MSCs *in vivo* (study 2).

Several protocols for differentiating cells into IPCs have been reported in iPS, ES, and MSCs. In this study, we performed a method similar to the previously reported method using human BM-MSCs [17], with modifications. This protocol involves three steps. In the first step, *β*-mercaptoethanol was used to induce the expression of the transcription factor Pdx-1, also known as insulin promoter factor 1. Pdx-1 is required for the early embryonic development of the pancreas and is also required for the subsequent differentiation [27]. The next step involves EGF and bFGF, which play an important role in cellular proliferation, differentiation, and survival [28]. EGF and bFGF have been reported to be useful in IPC differentiation [29, 30]. Exendin-4 can boost the expression of beta cell-related genes Pdx-1, Nkx2.2, Isl-1, and MafA [31]. Furthermore, IPCs generated from Wharton's jelly MSCs using a differentiated protocol with exendin-4 showed much better response to variable glucose levels [31]. The third step involves activin-A, betacellulin, nicotinamide, and hepatocyte growth factor. Many studies have used these substances to differentiate cells into IPCs *in vitro*. The combination of activin-A and betacellulin has been reported to enhance the proliferation into IPCs [32, 33]. Nicotinamide, which is a poly-ADP-ribose polymerase inhibitor, functions in the preservation of islet viability and function and represents a common extrinsic induction factor for MSCs [34]. HGF has been suggested to be a potent regulator of beta cell function and proliferation, and the effects of HGF are enhanced by activin-A [35, 36]. High glucose-containing medium was used throughout the differentiation protocol, because high glucose levels have been considered a potent inducer for pancreatic islet differentiation [29].

In our protocol for IPC differentiation, *μ*-pieces were used for three-dimensional cell culture. Several 3D culture materials have been established, such as Matrigel, scaffolds, and unattached culture dishes [37–39]. 3D cell culture supports tissue-specific function and physiological cell-cell and cell-matrix interactions. These factors contribute to various advantages for increasing cell viability, improving cell-type specific function and gene expression, and increasing cell secretion of proteins [40]. The advantage of *μ*-pieces, such as human recombinant peptide petaloid, was reported for islet cell culture and cell transplantation [41]. Spheroid-like cell aggregation-formed combination cells with *μ*-pieces can prevent central necrosis within cell aggregates, because nutrients and waste products can pass through the inner spheroid [42]. Our results showed that IPC generated from canine AT-MSCs in the 3D culture system showed better DTZ staining and insulin secretion compared with cells in conventional 2D culture dishes. These results were in agreement with a previous study that demonstrated that the 3D culture system using a collagen/hyaluronic acid scaffold enhanced the differentiation of IPCs from rat AT-MSCs [14]. Our findings demonstrated that canine AT-MSCs can be differentiated into IPCs using the protocol modified from human BM-MSCs under a 3D culture system.

We next performed functional evaluation of the IPCs from canine AT-MSCs *in vivo*. We first confirmed the improvement of the hyperglycemic state in diabetic mice after administration of the supernatant of IPCs differentiated from canine AT-MSCs. The blood glucose levels of all six mice in group IV (treated with the supernatant of IPCs) were significantly decreased compared with those of other groups. In an additional examination, all mice in group IV did not show improvement of the hyperglycemic state after administration with culture medium. This result demonstrated that insulin produced by canine IPCs decreased the blood glucose levels in STZ-induced diabetic mice.

Next, we assessed the effects of transplanted canine IPCs on STZ-induced diabetic SHO mice. After 15 days of observation, microvessels were observed on the surface of transplanted small clusters of IPCs. Histological examination revealed that small clusters of IPCs were engrafted in transplanted sites and included insulin-positive cells as identified by immunofluorescence. However, IPCs transplanted subcutaneously or intramesenterically did not improve the hyperglycemic states. There are several possible reasons for the failure to improve the hyperglycemic status after IPC transplantation. The first possibility may be that the number of transplanted IPCs is not enough for the donor's body size. We transplanted 1.5 × 10^6^ AT-MSCs according to a previous study that showed transplantation of IPCs differentiated from human BM-MSCs into STZ-induced diabetic rats [43]. The study demonstrated that intraperitoneal transplantation of 1.5 × 10^6^ BM-MSCs that differentiated into IPCs into diabetic rats led to the improvement of the hyperglycemic states. However, in a recent report on the transplantation of IPCs generated from human AT-MSCs with *μ*-pieces into diabetic mice, 4.8 × 10^7^ cells were transplanted under the kidney capsule or intramesentery region and led to normoglycemic states [44, 45]. One of these studies also calculated the cell number of generated IPCs and found that 1.8 × 10^4^ IPCs were obtained from 5 × 10^5^ AT-MSCs, indicating that 3.6% of the initial AT-MSCs differentiated into IPCs; thus, approximately 1.7 × 10^6^ IPCs are needed per mouse to gain the normoglycemic states in STZ-induced diabetic mice [45]. In our study, we did not calculate the differentiation ratio of IPCs, but the differentiation protocol referred to in our study reported approximately that 2.5% of human BM-MSCs differentiate into insulin-positive cells under a 2D conventional culture system [17]. These reports suggest that our cell numbers were too small to improve the hyperglycemic states in STZ-induced diabetic mice. Therefore, we will need to evaluate the quantitative differentiation efficiency in further study.

The second possible cause is the origin of the AT-MSCs. In our study, we collected adipose tissue from falciform ligament fat and isolated AT-MSCs. A previous study on the insulin secretion capacity of IPCs generated from human AT-MSCs reported that IPCs generated from visceral AT-MSCs showed poor differentiation and insulin secretion compared with those from subcutaneous AT-MSCs [45]. One reason why visceral AT-MSCs have been not suitable for differentiation into IPCs is that visceral AT-MSCs secrete more inflammatory cytokines such as IL-6 and TNF-*α* [46]. Multiple signalling cascades stimulated by inflammatory cytokines activate inducible nitric oxide synthase and result in apoptosis with impaired insulin biosynthesis and secretion. The study also indicated that IPCs generated from visceral AT-MSCs were inferior regarding glucose-stimulated insulin secretion. A stimulation index (SI), a parameter that reflects the insulin releasing function in response to glucose levels, of less than 3 does not normalize blood glucose levels after transplantation [45]. Our generated IPCs from canine visceral AT-MSCs showed increasing insulin secretion in response to high glucose levels, but the SI of our generated IPCs *in vitro* was only approximately 1.4 (calculated as the level of insulin concentration under high glucose condition (25 mM) divided by that under low glucose condition (5.5 mM)). Our result of glucose-stimulated insulin secretion ability was similar to that of human visceral IPCs (SI of subcutaneous IPCs was 3.8, but SI of visceral IPCs was 1.5) [45]. Therefore, our generated IPCs may not show sufficient insulin releasing function for improving the hyperglycemic state *in vivo*.

To differentiate cells into IPCs with more potent insulin secretion capacity, a more effective differentiation protocol will be needed. Other differentiation protocols containing histone deacetylase (HDAC) inhibitors such as trichostatin A and valproic acid have been reported [19, 47]. HDAC inhibitors induce a more open chromatin architecture and increased access for transcription factors. Moreover, HDAC inhibition was reported to be a potent driver of pancreatic cell lineage progenitors [48]. Therefore, we will need to evaluate other differentiation protocols to determine which protocol will be more suitable for canine AT-MSCs.

## 5. Conclusion

The results of this study demonstrated that the canine AT-MSCs can be differentiated into IPCs under a 3D culture system. To achieve stem cell-based therapy for canine DM for clinical use, further studies are required to establish a more effective differentiation protocol that induces increased insulin secretion *in vitro* and to determine the number of transplanted cells that are sufficient to improve hyperglycemic states *in vivo*.

## Data Availability

The data supporting the findings of this study are available from the corresponding author upon reasonable request.
